# Investigations of Zeolitic Materials at the NIST Center for Neutron Research

**DOI:** 10.6028/jres.106.048

**Published:** 2001-12-01

**Authors:** B. H. Toby

**Affiliations:** National Institute of Standards and Technology, Gaithersburg, MD 20899-8562 USA

**Keywords:** Crystallography, microporous materials, neutron diffraction, powder diffraction, zeolite

## Abstract

Crystallographic studies of four zeolitic materials using neutron powder diffraction data are presented. In most cases, these projects benefited from the combined use of neutron and x-ray measurements.

Dedication

This article is written in appreciation for the efforts of my colleagues Edward Prince, Judith Stalick, and Antonio Santoro. Combined, they have contributed nearly a century of service to NIST and have made many significant advances in neutron crystallography. Their accomplishments have made the work described here possible.

## 1. Introduction

Zeolites and related microporous materials have molecule-sized cavities. These materials have a wide range of applications, from consumer products to industrial processes. In the industrial arena, they are used as catalysts to produce gasoline and pharmaceuticals. Another application for these materials is the separation of N_2_ from O_2_, for both industrial and medical applications, as well as other types of gas separations. In our homes, zeolites are formulated in household detergents as ion exchangers that remove the calcium ions that make water “hard.” They are also used in double-pane windows to adsorb any intruding water that would otherwise fog the windows.

Zeolites are naturally occurring aluminosilicate minerals, but varieties of related zeolitic materials are now known, many of which do not have naturally occurring analogues. While some zeolitic materials are mined for commercial use, most are now synthesized.

Despite the wide range of uses, there are many basic facts about zeolites that are still poorly understood. One example is that why one zeolitic material may be preferable over another for a particular process is often not understood. Another example is that many different zeolitic materials can be formed using slightly different synthesis conditions, but the factors that change the products are frequently unknown. Determination of structures of zeolitic materials is important for understanding the properties of these materials, as well as for tailoring them to better suit a particular process. Crystallographic analysis of neutron powder diffraction data is often the primary technique for these studies, since understanding the siting of light atoms is central to this work and since these materials rarely form single crystals. This work is made more difficult by the relatively poor diffraction patterns seen from these materials, since they usually exhibit significant amounts of disorder. These patterns are usually weak compared to dense-phase materials; pores make up as much as 50 % of the volume of a zeolitic material.

Aluminosilicate zeolitic materials are constructed of tetrahedral [AlO_4_] and [SiO_4_] units that share O atoms, so that there are two O atoms for every Si or Al atom. The formal charges of the species for AlO_2_ and SiO_2_ are –1 and 0, respectively. This means that the presence of aluminum in the framework introduces a net negative charge that must be balanced by the presence of extra-framework cations. These cations create the ion-exchange capability of these materials and influence many other material properties. Because the cation locations often dictate the properties of the zeolite material, determination of cation siting is an another important facet of these structural studies.

Other related microporous materials have been created with substitution of all or some of the Al and Si atoms by a number of other elements, such as B, Be, Cr, Fe, Ga, Ge, Mn, P, Ti, and Zn. These elements also accept tetrahedral coordination and the formal charges on the TO_2_ species are usually neutral or −1.

Neutron diffraction studies of zeolites have been a focus area at the NIST Research Reactor for nearly two decades [1]. As discussed previously, the BT-1 instrument offers the best instrumental resolution in the U.S. for neutron crystallography [2]. The upgrade of the 75 take-off angle monochromator to Ge(311) in recent years has further improved the instrument for zeolite studies by improving resolution at lower angles and increasing the sensitivity by a factor of two. The instrument is heavily utilized and approximately one thousand complete diffraction patterns are collected each year, on hundreds of different materials. A significant fraction of this work pertains to zeolitic materials. The following will describe some of the more recently published work. In most cases, both neutron and x-ray diffraction results were utilized. This article will explore some of the advantages gained through use of these complementary tools.

## 2. Microporous Lithosilicates

Only recently has a new family of microporous materials, constructed from tetrahedrally coordinated Li and Si, been discovered [3]. The use of Li as a tetrahedral species is an important development, as the [LiO_4_] unit is more flexible than other tetrahedral building units and thus can create structures that would be highly strained with other tetrahedral species. In addition, the high charge on the LiO_2_^3−^ formula unit requires a large number of charge-balancing cations, increasing the possible utility of these materials. The first complete structural characterization of a microporous lithosilicate was recently performed using data from the BT-1 instrument [4].

**Fig. 1:**
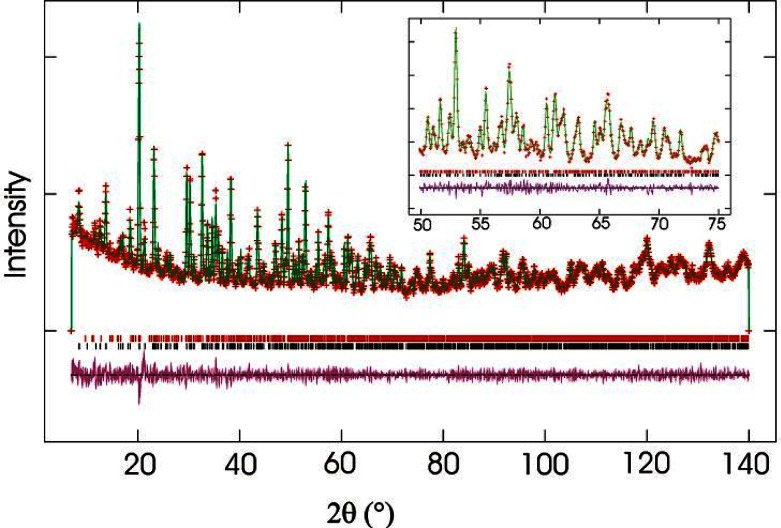
Observed neutron diffraction intensities (crosses) for deuterium-exchanged RUB-29 superimposed on those computed from the crystallographic model (upper line). The upper and lower sets of short vertical lines indicate reflections of the impurity compound RUB-23 and RUB-29, respectively. The lower curve shows the difference between the observed and calculated data. The plot is enlarged in the high angle range between 50° < 2*θ* < 75° to emphasize agreement between the data and the model.

**Fig. 2:**
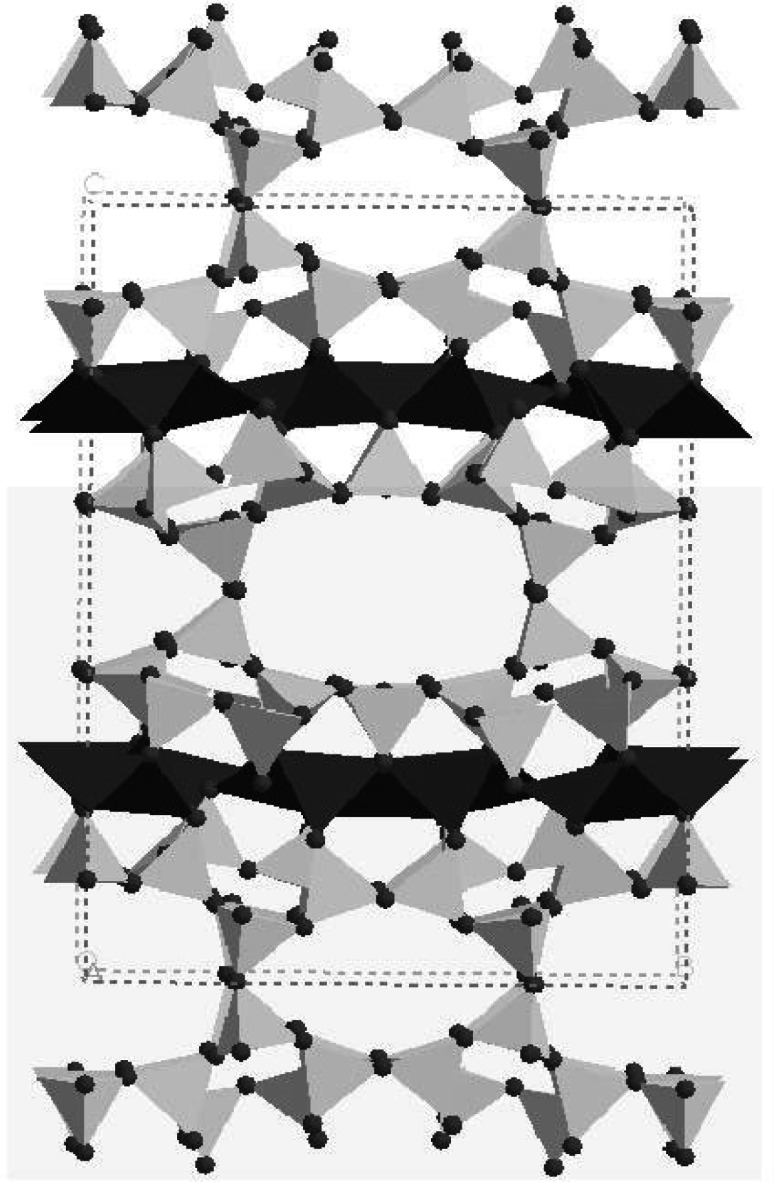
The framework structure of RUB-29, viewed along the *a* axis. Si tetrahedra are shaded as light gray while dark shading is used for Li.

The structure of RUB-29 was determined from a 10 × μm × 10 μm × 2 μm single crystal using synchrotron x rays [4]. The material has 13 crystallographically unique tetrahedral atoms, as well as 22 oxygen atoms, and thus is one of the most structurally complex zeolitic materials reported to date. It would have been extremely difficult, if not impossible, to determine this structure without this x-ray single-crystal data. Neutron powder diffraction data were still needed to confirm the siting and occupancy of the Li atoms in the framework since the average number of electrons in a site determines x-ray scattering intensities. Thus with x rays, scattering from a light atom at full occupancy is virtually indistinguishable from that of a heavier atom at low occupancy. Further, x rays are relatively insensitive to light atoms. Neutron powder diffraction data collected on 1 g of the sample, along with a fit to these data, are shown in Fig. 1. From the neutron data, the sites for the four Li framework atoms were determined, along with four non-framework Li sites and seven other cation/water sites. The framework is shown in Fig. 2. This work demonstrates that even when single-crystal x-ray studies can be performed, there is still significant advantage to combining these measurements with neutron powder diffraction data.

## 3. Zeolite RHO

**Fig. 3:**
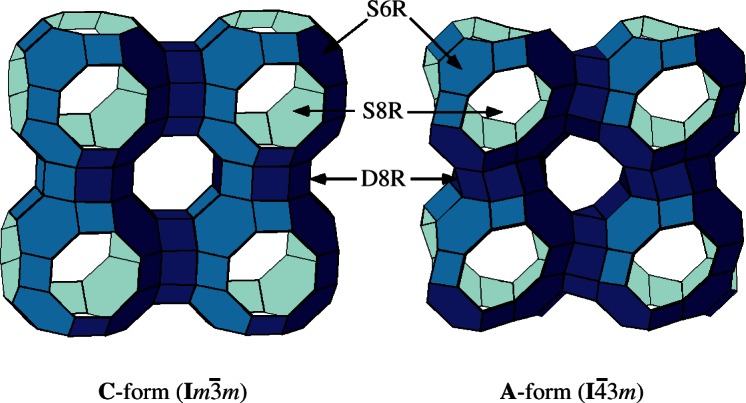
Zeolite RHO in the C (centric, *Im* 3*m*) and A (acentric, *I* 43*m* 3m) topologies. Vertices represent tetrahedrally coordinated atoms; oxygen atoms are omitted to improve figure clarity.

Zeolite RHO is known to undergo significant changes in structure as a function of temperature and cation exchange. Examples of this are shown in Fig. 3. Another new class of zeolitic materials, microporous aluminogermanates, was recently discovered [5,6]. One aluminogermanate material has the RHO topology. This discovery prompted a comparison of Li siting in an aluminosilicate Li-RHO with that in an aluminogermanate Li-RHO material. A neutron powder diffraction study on this material revealed that Li cations bind in a site in the RHO framework that has not occupied in any other RHO material. The difference in siting is attributed to the difference in Ge-O bond distances and Ge- O-Al flexibility in comparison to those in aluminosilicates. This demonstrates how cation siting in zeolitic materials is determined by framework bond distances and flexibility [7].

Another property of zeolite RHO, the migration of cations between sites with temperature, has been explored using *in situ* x-ray and neutron diffraction measurements. Cations such as Cd may site in rings that block the access to the material’s pores. At elevated temperatures, these cations migrate to other sites, allowing guest species into the zeolite pores. This migration can give rise to what has been called a “trap door” effect: when the temperature is lowered, the cations return to their original positions and trap the guest species in the pores [8,9]. We have recently demonstrated that cations return to their original sites only when the zeolite is cooled in the presence of minor amounts of water [10,11]. Neutron diffraction explains this, by revealing the presence of water bound to the cation under conditions where the zeolite was previously believed to be dehydrated. The heating process removes this water and the cation migrates to a site where higher coordination can be achieved. The cation returns to occupy the “low temperature” site only when water is present to bind to the cation and increase the coordination at this site. If the material is kept in a water-free environment, the cation remains in the “high temperature” site even after the temperature is lowered. These results also call into question the observation of negative thermal expansion in Sr-RHO [12]. It is now believed that the unit cell contracts upon heating, due to a reversible loss of water.

## 4. Modifying Pore Sizes in ETS-4

The titanosilicate ETS-4 is composed of tetrahedral [SiO_4_] and both tetrahedral [TiO_4_] and octahedral [TiO_6_] building units [13]. This material crystallizes with planar defects, called stacking faults, which disrupt access to the pores, but only in certain directions. The result is that while the framework structure might indicate otherwise, the pore access in ETS-4 occurs only via rings of eight tetrahedral atoms, called eight-rings.

ETS-4 is prepared in hydrated form and like many zeolitic materials can be dehydrated by heating. Many microporous materials dehydrate reversibly; others decompose forming non-porous phases. Unusually, ETS-4 loses some of its long-range crystallinity during dehydration, but retains much of the original microporosity. This dehydration is usually irreversible.

**Fig. 4:**
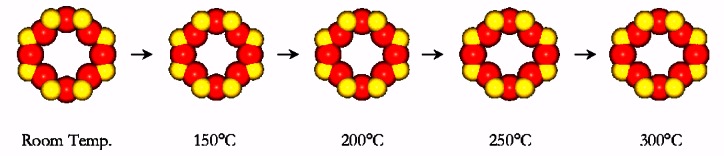
Size and shape of the eight-ring pore opening in ETS-4 as a function of dehydration temperature.

**Fig. 5:**
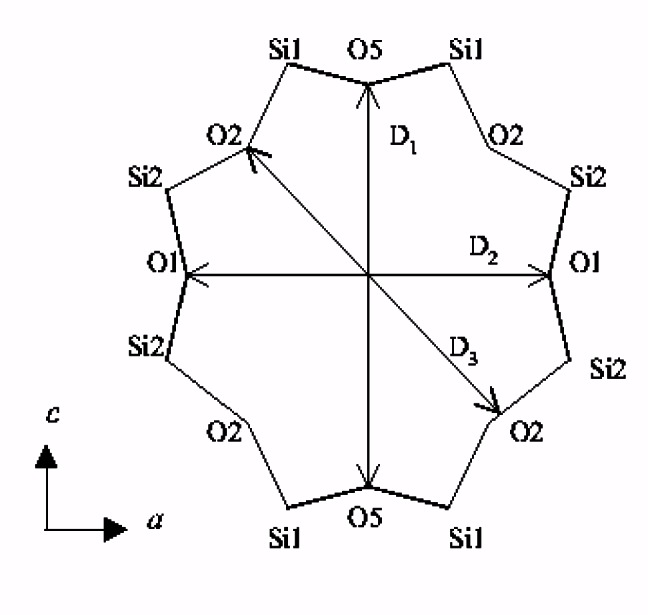
Atom and pore-opening distance labeling scheme in the eight-ring opening of ETS-4, as used in Table 1.

**Fig. 6:**
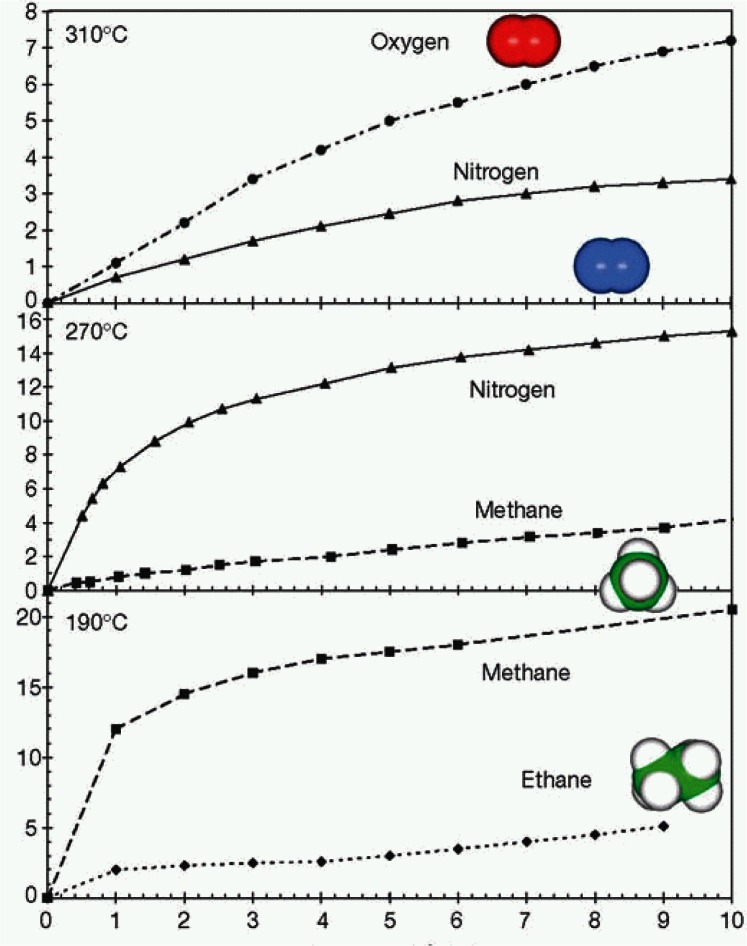
Isotherms for ethane, methane, nitrogen and oxygen adsorption as a function of dehydration temperature for the ETS-4 material. The horizontal axis is the partial pressure of the selected gas in a He carrier gas (arbitrary units) and the vertical axis is the amount of gas adsorbed (ml/g).

Since O atoms have poor x-ray scattering power, compared to neutrons, neutron diffraction is able to determine O-O distances with greater precision. Results from neutron diffraction measurements performed at BT-1 are shown in Figs. 4, 5, and Table 1 [14]. These results show how the eight-ring diameter changes as a function of dehydration. This effect allows the pore size for the material to be adjusted to admit some molecules while excluding others. Examples of separations performed using ETS-4 are shown in Fig. 6, which shows the relative uptake of different molecules as a function of their partial pressure in a gas mixture. The precision of the pore size adjustment is demonstrated by the difference in uptake of O_2_ versus N_2_, shown at the top of this figure. Note that the van der Waals radii of these two gases differ by only approximately 0.1 Å.

## 5. Structure-Directing Agents in CIT-1

Two borosilicate zeolitic materials, SSZ-33 and CIT-1, have related structures and have been designated the structure code CON by the Structure Commission of the International Zeolite Association [15]. Unlike CIT-1, which is nearly free of defects, SSZ-33 has a very high density of stacking faults (≥30 %) [16]. These materials, like many other zeolitic materials, are synthesized in the presence of a sacrificial organic cation, called a structure directing agent (SDA) that is trapped in the pores during synthesis. To free the pore spaces, the SDA is oxidized away by calcination in air.

**Fig. 7:**
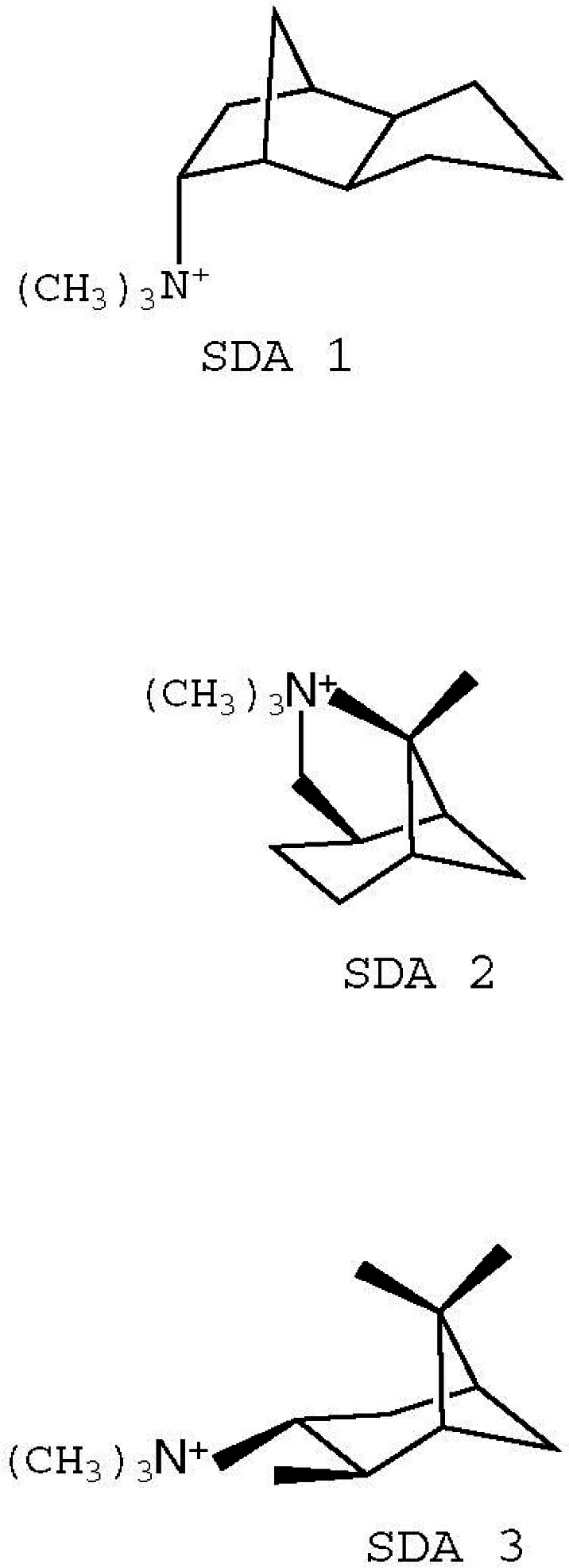
Molecular diagrams for the three structure-directing agents, SDA 1, SDA 2, and SDA 3 investigated for CIT-1 synthesis.

Several organic cations, shown in Fig. 7, were investigated with respect to CON family materials. Species SDA 1 can be used to synthesize SSZ-33, while, only SDA 2 has been found to synthesize CIT-1, without significant stacking faults. Interestingly, cation SDA 3, while quite similar to SDA 2, cannot be used to prepare any material in the CON family.

To better understand the SDA-framework interactions, the location of the SDA cation was determined [17]. Location of disordered guest molecules in zeolitic pores is a difficult crystallographic problem. For this reason, a simultaneous Rietveld fit to both neutron powder diffraction data and synchrotron x-ray powder diffraction data was performed using an “as-synthesized” CIT-1 sample where SDA 2 was not removed. Use of two types of diffraction data increases the effective number of observations, which allows more detailed crystallographic modeling. In this work, selective deuteration of the methyl groups of the quaternary nitrogen enabled the orientation of this group to be determined. To prove that the crystallographic result is the only plausible structural model for these data, molecular modeling used to find all possible sites for the SDA cation. These sites were investigated systematically to prove the uniqueness of the crystallographic result.

**Fig. 8:**
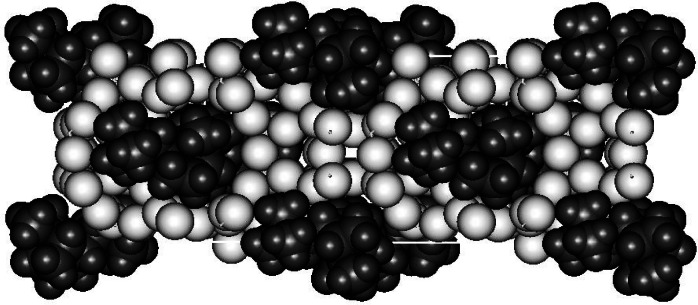
The minimum energy structure for SDA 2 in CIT-1, viewed though the 10-ring pores. The SDA cations are shown as dark spheres while the framework O atoms are shown as lighter spheres. Atoms are shown with their van der Waals radii, 1.55 Å, 1.1 Å, and 1.35 Å, for C, H and O, respectively.

The final crystallographic model, shown in Fig. 8, shows a very tight fit between the SDA ions and the CIT-1 framework, but also confirms that four SDA cations can pack into the pore space of a single unit cell. This had not been predicted. Rather, our initial Monte-Carlo molecular modeling indicated that only three SDA cations could be accommodated in the CIT-1 pores. Later molecular modeling work, starting from the crystallographic model, confirmed that four molecules of either SDA 1 or 3 can pack in the CIT-1 pores without any unfavorable energetic interactions. However, SDA 3 could not pack as effectively in the same space, explaining why SDA 3 does not synthesize the CON framework. Molecular modeling of SDA packing in the presence of stacking faults showed no significant differences between SDA 1 and 2, indicating that these stacking faults likely arise as a kinetic effect rather than due to thermodynamics.

## 6. Conclusions

Structural studies of zeolitic materials are demanding, due to the complexity of the materials, as well as their poor scattering abilities. Nonetheless, by maximizing the number of observations, through use of high resolution and high signal-to-noise diffraction measurements from both neutron and synchrotron instruments, more complex structures can be determined. These results bring considerable insights to these interesting and valuable materials.

## Figures and Tables

**Table 1:** The size of the eight-ring opening in ETS-4 as a function of dehydration temperature. Distances are the separation between opposite O5 (D_1_), O1 (D_2_) and O2 (D_3_) atom pairs (see Fig. 5), adjusted for the van der Waals radius of O

van der Waals opening (Å)	Dehydration temperature, °C				
	RT	150	200	250	300
*D*_1_	4.27	3.97	3.95	3.94	3.90
*D*_2_	4.43	4.02	4.09	4.57	4.57
*D*_3_	3.61	3.28	3.29	3.27	2.77
